# The Finnish Cardiovascular Study (FINCAVAS): characterising patients with high risk of cardiovascular morbidity and mortality

**DOI:** 10.1186/1471-2261-6-9

**Published:** 2006-03-03

**Authors:** Tuomo Nieminen, Rami Lehtinen, Jari Viik, Terho Lehtimäki, Kari Niemelä, Kjell Nikus, Mari Niemi, Janne Kallio, Tiit Kööbi, Väinö Turjanmaa, Mika Kähönen

**Affiliations:** 1 Department of Pharmacological Sciences, Medical School, University of Tampere, Finland; 2 Department of Clinical Physiology, Tampere University Hospital and Medical School, University of Tampere, Finland; 3 Tampere Polytechnic – University of Applied Sciences, Finland; 4 Ragnar Granit Institute, Tampere University of Technology, Finland; 5 Laboratory of Atherosclerosis Genetics, Department of Clinical Chemistry, Tampere University Hospital, Finland; 6 Centre for Laboratory Medicine, Medical School, University of Tampere, Finland; 7 Heart Centre, Department of Cardiology, Tampere University Hospital, Finland

## Abstract

**Background:**

The purpose of the Finnish Cardiovascular Study (FINCAVAS) is to construct a risk profile – using genetic, haemodynamic and electrocardiographic (ECG) markers – of individuals at high risk of cardiovascular diseases, events and deaths.

**Methods and design:**

All patients scheduled for an exercise stress test at Tampere University Hospital and willing to participate have been and will be recruited between October 2001 and December 2007. The final number of participants is estimated to reach 5,000. Technically successful data on exercise tests using a bicycle ergometer have been collected of 2,212 patients (1,400 men and 812 women) by the end of 2004. In addition to repeated measurement of heart rate and blood pressure, digital high-resolution ECG at 500 Hz is recorded continuously during the entire exercise test, including the resting and recovery phases. About 20% of the patients are examined with coronary angiography. Genetic variations known or suspected to alter cardiovascular function or pathophysiology are analysed to elucidate the effects and interactions of these candidate genes, exercise and commonly used cardiovascular medications.

**Discussion:**

FINCAVAS compiles an extensive set of data on patient history, genetic variation, cardiovascular parameters, ECG markers as well as follow-up data on clinical events, hospitalisations and deaths. The data enables the development of new diagnostic and prognostic tools as well as assessments of the importance of existing markers.

## Background

Coronary heart disease (CHD) is the leading cause of morbidity and mortality in industrial countries. The exercise stress test is commonly used for diagnosing CHD. The first and most widely applied electrocardiogram (ECG) marker used to indicate ischemia during exercise is depression of the ST segment [1], but several other parameters have been introduced to complement the information received. Recently, some of these ECG markers have also been proposed to serve as prognostic means for predicting the clinical course of CHD [2,3]. Moreover, exercise capacity as well as the levels of and changes in blood pressure and heart rate (HR) during the exercise test have been suggested to predict the incidence of cardiac events and deaths [2,4-10]. However, the results so far are not conclusive, but rather they justify further study and research to find novel exercise test parameters for both diagnostic and prognostic purposes.

Genetic variation has been shown to affect the cardiac ion channels [11,12], the sympathetic nervous system [13,14], function of arteries and veins [15,16], regulation of blood pressure [17,18] as well as the cardiac muscle and contractility [19]. Consequently, hereditary differences probably explain part of the inter-individual variation in cardiovascular responses during short- and long-term physical exercise [20]. However, few large and systematic studies have been performed on the interaction of genes with physiological and pathophysiological cardiovascular responses during exercise [21]. Heavy exercise also induces pathological cardiac disorders, such as arrhythmia, in susceptible persons [22,23]; the possible underlying hereditary factors are poorly known. Furthermore, to our knowledge, no studies have been published on the interactions between genes and exercise regarding cardiovascular mortality.

The effects of many drugs commonly used for the treatment of hypertension, CHD and other cardiovascular disorders vary widely from one person to another. Part of this variation has been attributed to genetic polymorphisms in factors contributing either to pharmacokinetics or pharmacodynamics – i.e., to pharmacogenetics [24-26]. Since a majority of patients with cardiovascular illnesses use one or several types of these drugs, we hypothesise that exercise and the polymorphisms with pharmacogenetic influence might also have interactions affecting the physiological parameters measured during a clinical exercise test.

The Finnish Cardiovascular Study (FINCAVAS) was designed and is being conducted to find exercise test markers predicting future cardiovascular events and deaths. Many of the present patients undergo coronary angiography, and the database gives grounds for improving the diagnostic and prognostic accuracy of the exercise test by determining associations between ECG, haemodynamic parameters and angiographic findings. Moreover, we aim to elucidate extensively the effects and interactions of exercise, commonly used cardiovascular medications and a variety of candidate genes in people without cardiac diseases as well as in patients with CHD. As a corollary to these, the purpose of FINCAVAS is to construct a risk profile to identify individuals at a high or low risk of cardiovascular diseases and events.

## Methods and design

### Study cohort

The participant pool consists of patients who undergo exercise stress tests at Tampere University Hospital. All the consecutive patients coming in for an exercise stress test and willing to participate in the study have been and will be recruited between October 2001 and December 2007. Our goal is to recruit roughly 5,000 patients. Currently, the research group is actively analysing the data on the 2,212 patients (1,400 men and 812 women) recruited by the end of 2004. The study protocol was approved by the Ethical Committee of the Hospital District of Pirkanmaa, Finland, and all patients have given informed consent prior to the interview and measurements as stipulated in the Declaration of Helsinki.

In addition, roughly one thousand patients scheduled for spiroergometer testing (SensorMedics VMax29c, VIASYS Healthcare Inc., Yorba Linda, CA, USA) with pre- and post-test spirometry will be recruited between December 2005 and the end of 2007.

No specific sample size calculations were made prior to study initiation. In general terms, if we want to find a 10% difference in the prevalence of certain genotypes with a significance level of p < 0.05 and to reach an 80% ability to find significant differences between the groups, we need hundreds of patients. Since the study population is often divided into subgroups based on, for example, age, sex and medication, the size of the cohort should preferably be at least one thousand.

### Study flow

After an informed consent has been signed, the medical history of each patient is gathered with a computer-based questionnaire form [27]. Thereafter, the exercise test is performed. Blood samples are drawn for DNA analyses. Some of the patients are examined with coronary angiography either before or after the exercise test.

### Exercise test protocol

Prior to the exercise stress test, the subject lie down in supine position for 10 minutes, and the resting ECG is digitally recorded. The exercise test is performed using a bicycle ergometer with electrical brakes. The lead system used is the Mason-Likar modification of the standard 12-lead system [28]. The initial workload varies from 20 W to 30 W, and the load is increased stepwise by 10–30 W every minute. During the test, HR is continuously registered with ECG, while the systolic (SAP) and diastolic (DAP) arterial pressures are measured with a brachial cuff every two minutes. Breathing frequency is measured and the patient is asked about the perceived exertion level every two minutes using Borg's scale with 15 categories [29].

Most of the exercise tests are sign- and symptom-limited maximal tests using the recommended criteria for termination, but some of the tests for evaluating the status after a myocardial infarction (MI) have an HR upper limit of 120–130 bpm. The recovery phase after the exercise is at least five minutes.

### Laboratory analysis and DNA extraction

FINCAVAS has access to the laboratory tests performed on the study patients at Tampere University Hospital between October 1998 and the end of the study. Plasma triglycerides, total and high-density lipoprotein (HDL) cholesterol as well as sensitive C-reactive protein (CRP) concentrations are analysed by a Cobas Integra 700 automatic analyser with reagents and calibrators recommended by the manufacturer (Hoffmann-La Roche Ltd., Basel, Switzerland). Low-density lipoprotein (LDL) concentration is calculated using Friedewald's formula [30]. A typical detection limit for CRP is 0.25 mg/l [31]. Genomic DNA is extracted from peripheral blood leukocytes using a commercially available kit and Qiagen BioRobot M48 Workstation according to the manufacturer's instructions (Qiagen Inc., Hilden, Germany).

### Polymorphisms, genotyping and the selection of candidate genes

One of the central objectives of FINCAVAS is to examine the effects of polymorphisms known or suspected to alter the development of atherosclerosis, cardiovascular function, pathophysiology or related pharmacogenetics. Moreover, we aim to find new polymorphisms affecting these processes. In the first of our two genotyping strategies, the genes found to be expressed in the arterial wall – thus possibly directly affecting the development of atherosclerosis – are selected for the detailed genotype-haplotype analysis. The selection is based on the results of another ongoing project in which the expression of the entire genome, roughly 30 000 genes, is scanned from 30 freshly collected arterial tissue samples with morphometrically and immunohistochemically verified atherosclerotic lesions.

The expression analysis is performed using Illumina BeadArray™ technology (Illumina, Inc, San Diego, CA, USA). After selecting the genes statistically most significantly expressed in the arterial wall, we design an Illumina custom panel for up to 1536 markers for single nucleotide polymorphisms of the genes chosen. The advantage of this strategy is the low price per sample, which enables us to analyse all the participants of FINCAVAS. As the price of genotyping steadily decreases, we are technically equipped to perform even more genotyping using the Illumina BeadChip arrays.

In the second strategy, genetic variation in the following cascades and anatomical structures particularly relevant for FINCAVAS are studied: regulation of blood pressure, adrenergic signal transduction, cardiac ion channels as well as pharmacokinetics and pharmacodynamics of drugs used to treat hypertension and CHD.

In this strategy, DNA samples are genotyped by employing the 5' nuclease assay and fluorogenic allele-specific TaqMan MGB probes [32], using the ABI Prism 7900HT Sequence Detection System (Applied Biosystems, Foster City, CA, USA). The samples are pipetted using an automated TECAN Freedom EVO-100 pipetting robot (Tecan Group Ltd, Männedorf, Switzerland). The nucleotide sequences of the primers and probes used in the polymerase chain reaction (PCR) are deduced from published sequences deposited in the GenBank and Celera databases and synthesised by Applied Biosystems. The PCR reactions containing genomic DNA, 1 × Universal PCR Master Mix, 900 nM of each primer and 200 nM of each probe are performed in 384-well plates using the standard protocol in a total volume of 5 μl.

End-point fluorescence is measured and genotype calling carried out by the allelic discrimination analysis module after the PCR results in the identification of polymorphisms chosen. Negative and positive controls (known genotypes) and random duplicates are used as quality control. Genotypes are analysed in the Centre for Laboratory Medicine, Department of Clinical Chemistry, Laboratory of Atherosclerosis Genetics at Tampere University Hospital.

### Follow-up

**Figure 1:**
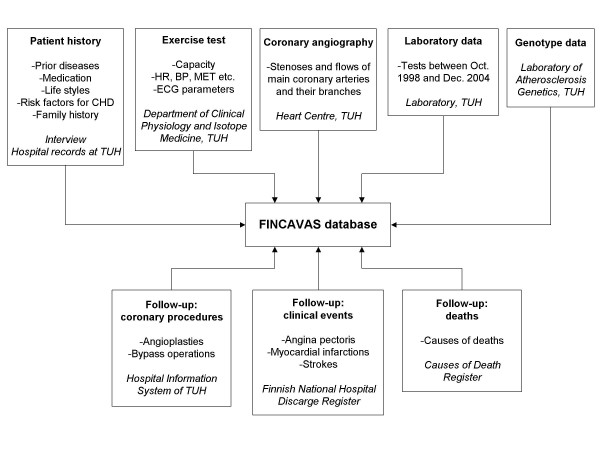
Data sources utilised in the present study. CHD, coronary heart disease; HR, heart rate; BP, blood pressure; MET, metabolic equivalent; ECG, electrocardiogram; TUH, Tampere University Hospital.

The follow-up data consists of information on major cardiovascular events (hospitalisation due to angina pectoris, MI or stroke), coronary procedures (angioplasties and bypass operations) and causes of deaths (Fig. 1). The follow-up data will be gathered at 2, 5 and 10 years. Information on clinical events will be collected from the Finnish National Hospital Discharge Register and data on mortality from the Causes of Death Register; these sources have been shown to be reliable [33]. Information on operative procedures will be received from the Hospital Information System of Tampere University Hospital.

### Phenotype definitions

The presence of CHD in the patients included in our database can be defined in several ways. First, the existence of CHD prior to the exercise test is based on patient interviews, resting ECG recordings and existing hospital records. Secondly, the exercise test *per se *provides information on CHD diagnosis; this can be combined with the pre-test probability of CHD using Bayesian logic. Thirdly, some 20% of the patients have undergone coronary angiography that unambiguously reveals the status of coronary arteries; different stenosis criteria for CHD can be established for different purposes. The CHD definition used in each sub-project depends on the specific aims of the project.

History of prior MI is based on patient interviews, resting ECG recordings and hospital records. Data on the existence of hypertension, valvular diseases, cardiomyopathies, other heart diseases and diabetes (types 1 and 2) is based on interviews and hospital records. The presence of abnormal atrio-ventricular conduction, right (RBBB) and left (LBBB) bundle branch blocks, delta waves, pathological Q waves and long QT syndrome are based on resting ECGs and hospital records.

### Data architecture

The material in the FINCAVAS database is woven together from many data sources (Fig. 1). The patient history data is based on both patient interview and hospital records; the data is recorded with a computer-based form [20]. Data on demographics (i.e., age, sex, weight, height, body mass index, waist to hip ratio, occupation) and lifestyle (alcohol consumption, physical exercise at work and leisure time) is collected using the same software before the exercise test. Furthermore, exposure to classic cardiovascular risk factors (i.e., smoking, hypertension, diabetes, occurrence of hyperlipidemia and family history) is covered extensively.

Cardiovascular symptoms (including the functional capacity classification developed by the New York Heart Association, NYHA) and diseases are recorded in detail, with special emphasis on CHD and prior MIs. All the medications used by the patient are entered into the database, including the length of possible pause before the exercise test.

The actual exercise tests generate the majority of the data. Values for HR, SAP, DAP, breathing frequency, physical load, perceived exertion level, peak expiratory flow before and after the exercise as well as oxygen saturation in a finger tip, if measured, are gathered on the software mentioned earlier.

Continuous ECG is recorded at 500 Hz with CardioSoft exercise ECG system (Version 4.14, GE Healthcare, Freiburg, Germany) and analysed by Modified CASE software (GE Healthcare, Freiburg, Germany). The system recognises ventricular escape beats, premature ventricular complexes (PVC), premature supraventricular complexes, pauses of 1 or 2 missed beats, ventricular bigeminy, ventricular couplets (2 PVCs), ventricular runs (> 2 PVCs), ventricular tachycardia, atrial fibrillation/flutter, ventricular fibrillation and asystole. In addition, the following parameters are assessed and recorded: amplitudes of P, Q, R, S and T; durations of P, Q, R, S, RP and QT; levels of J-point and ST-segments at 60 ms; ST integral; ST slope; T wave alternans; ST-segment depression/heart rate (ST/HR) index; ST/HR slope; ST/HR hysteresis [34,35] and, finally, HR recovery and noise level. These parameters are determined from 14 ECG leads: aVL, I, -aVR, II, aVF, III, V1 to V6, CM5 and V4R. Furthermore, all these parameters are determined at 14 points in time: before exercise, seven times during the exercise (start, 2 min, 4 min, 6 min, 8 min, 10 min and maximal load), and six times during the rest after the exercise (1 min, 2 min, 3 min, 4 min, 5 min and 6 min). Therefore, more than 3,500 ECG parameters are determined for each exercise test and stored into a separate database. However, any other points in time besides the ones specified here as well as additional parameters can be analysed as needed based on the continuous digital ECG recordings.

Raw data on laboratory tests is received from Tampere University Hospital. This bulk of data covers all the laboratory tests performed on the study patients. Minimum and maximum values for each test on each patient are searched using database queries and recorded into the FINCAVAS database.

The data on operative procedures is collected from the Hospital Information System of Tampere University Hospital. For each patient, an angiography is linked to an exercise test if they were a maximum of 6 months apart, and if no coronary bypass operations or coronary angioplasties are performed between them.

All the coronary angiographies are analysed by the same dedicated cardiologist (MN). TIMI (thrombolysis in myocardial infarction) flow, diameter and %-stenosis in the ostial, proximal, middle and distal parts of the left main (LM), the left anterior descending (LAD), the left circumflex (LCX), and the right (RCA) coronary arteries are registered. Furthermore, the same parameters are assessed for major branches: the diagonal branches I and II as well as the first septal branch of the LAD; the left obtuse marginal I and II as well as posterior descending branches of the LCX; posterior descending, right ventricular and posterior left ventricular branches of the RCA; and, finally, the left intermediate marginal branch. The stenoses are graded into seven categories: 0%, <50%, 50–74%, 75–89%, 90–98%, 99% and 100%.

With the exception of ECG parameters, the data depicted above is combined into a single SPSS file (release 12.0.1 for Windows, SPSS Inc, Chicago, Illinois, USA). The file comprises of 510 variables. Due to the enormous amount of ECG data, the ECG parameters are stored in separate files and will be added to the SPSS file based on the requirements of each subproject.

### Data validation

Laboratory and ECG data are entered into source databases or data files electronically and automatically. The other sources necessitate manual input. However, transferring the data from separate databases to the FINCAVAS database is done electronically using database tables and queries. Adequate cross-checks are done to ensure the accuracy of the data. Furthermore, data is validated by checking missing data and the plausibility of the ranges of values, as well as by inspecting the consistency of related data fields. Errors identified by these procedures result in checking the raw data when possible or eliminating the incorrect piece of data.

## Discussion

FINCAVAS is aimed at characterising individuals with a high risk of cardiovascular diseases and death. By the end of 2004, data had been gathered on more than 2,200 patients. The recruitment period will continue until the end of 2007, and the final number of patients is estimated to reach 5,000. All of the participants will go through detailed interviews and a clinical exercise test. Moreover, approximately 20% of the patients will be examined with coronary angiography, and 5–10% will perform another exercise test during the study period. The high number of exercise tests combined with other related data is one of the particular strengths of this study. To our knowledge, this is the first large-scale database with digitally recorded high resolution ECG data covering the entire duration of the exercise test.

Traditional ECG parameters for CHD diagnosis during exercise test – such as the amount and type of depression of the ST segment as well as T wave changes – are reasonably accurate in patients with an intermediate pre-test likelihood for having disease [36,37]. However, the test lacks sensitivity and specificity for many patient groups, particularly among women [38]. A central objective of FINCAVAS is to enhance the accuracy of exercise ECG in diagnosing CHD by combining some of the existing markers and by creating completely new algorithms. The digital high-resolution exercise ECG of the patients undergoing a coronary angiography is a valuable and fundamental resource for this purpose.

Several studies have demonstrated exercise capacity as an independent predictor of cardiac events and mortality in healthy persons and those with cardiac diseases [2,7-9,39]. The HR profile during exercise and recovery is a predictor of death [5,6,10]. Furthermore, the changes in blood pressure during and after exercise have been shown to predict cardiovascular events and mortality [4,40]. However, the importance of various combinations of the predictive parameters is poorly known. When it comes to ECG parameters, ST depression [2] and ST/HR analysis [3] during exercise have been connected to survival, but no studies thus far have revealed whether the values of and changes in T wave alternans, ST/HR hysteresis [34,35], HR variability and many other ECG parameters during exercise are linked with mortality. Our research team has made extensive and persistent efforts in studying the sophisticated CHD diagnostics based on ECG parameters and ECG leads [34,41-44]. The present continuous ECG at 500 Hz during the entire exercise test provides a solid foundation for testing the relevance of both existing prognostic parameters and those developed during the project.

The importance of classical risk factors for atherosclerosis has been evaluated in detail. In recent years, many genetic polymorphisms have been associated with atherosclerosis [45,46]. However, the majority of these new risk factors provide limited contribution to the total risk of cardiovascular morbidity and mortality. Therefore, defining and measuring specific and precise phenotypes is of crucial value when the quantitative importance of these risk factors and markers are assessed.

Some of the phenotype data included in FINCAVAS, such as angiographic findings and ECG parameters, can be considered particularly accurate. Blood pressure and breathing parameters during the exercise test are also accurate, while medical history may constitute the data most vulnerable to imprecision. Knowledge of the pharmaceuticals used and prior diseases are collected with care, but the process inherently bears some level of uncertainty. Many middle-aged and elderly individuals are suffering from considerable atherosclerosis and CHD although they may remain undiagnosed. Even MI, the most serious complication of CHD, is often silent and diagnosed only when a routine ECG is recorded. The level of uncertainty for each phenotype needs to be taken into account when the results of analyses and classifications using these parameters are interpreted.

Many polymorphisms are most likely not disease-causing genes, but rather risk modifiers, and might thus also influence the progression of cardiovascular diseases. Although certain polymorphisms do not induce marked effects on cardiovascular parameters, they may still have linkage effects with some functionally more important polymorphisms. In the course of FINCAVAS, the independent and combined effects of many genotypic variations on cardiovascular morbidity and mortality will be assessed prospectively with a long-term follow-up. The purpose of this strategy is not only to study the effects of polymorphisms on known risk factors, but also to find new mechanisms and factors contributing to the aetiology of cardiovascular diseases directly in the arterial wall. To achieve this goal, we will exploit and use high throughput techniques for expression analysis and genotyping to find genes affecting the expression, synthesis and function of proteins associated with cell signal transduction, regulation of inflammation, apoptosis and lipid metabolism directly in the cells of the arterial wall. Better understanding of the function of the genes in these cells during the progression of cardiovascular disease could serve as a new tool in both research and therapeutic applications.

FINCAVAS is subject to certain limitations. We include all patients scheduled for a clinical exercise test at a university setting, which is both a strength and a weakness of the study. The present study population consists of patients with a wide spectrum of ages, life styles, histories and status of cardiac diseases. It is probable that the importance of many genotypes, responses to exercise and ECG markers vary from one patient group to another. Therefore, the study enables the assessment of risks for many patient groups, but, on the other hand, these groups need to be adequately recognised before analyses. In some cases, the subgroups may still be relatively small in spite of the high total number of patients.

In conclusion, FINCAVAS is compiling an extensive set of data on patient history, genetic variation, cardiovascular parameters and ECG markers prior to, during and after an exercise test. Furthermore, approximately 20% of the patients are being examined with coronary angiography. As the follow-up progresses, this data will enable us to estimate the importance of different diagnostic and prognostic markers for many distinct patient groups.

## Competing interests

The author(s) declare that they have no competing interests.

## Authors' contributions

TN drafted the first version of the manuscript. TN, RL, JV, TL, KaN, VT, TK, KjN, MN, JK and MK participated in the design of the study. JK and TN have implemented the software used in the study. All authors read and approved the final manuscript.

## Pre-publication history

The pre-publication history for this paper can be accessed here:



## References

[B1] Stern S (2002). State of the art in stress testing and ischaemia monitoring. Card Electrophysiol Rev.

[B2] Myers J, Prakash M, Froelicher V, Do D, Partington S, Atwood JE (2002). Exercise capacity and mortality among men referred for exercise testing. N Engl J Med.

[B3] Bigi R, Cortigiani L, Gregori D, Bax JJ, Fiorentini C (2005). Prognostic value of combined exercise and recovery electrocardiographic analysis. Arch Intern Med.

[B4] Mundal R, Kjeldsen SE, Sandvik L, Erikssen G, Thaulow E, Erikssen J (1996). Exercise blood pressure predicts mortality from myocardial infarction. Hypertension.

[B5] Jouven X, Empana JP, Schwartz PJ, Desnos M, Courbon D, Ducimetiere P (2005). Heart-rate profile during exercise as a predictor of sudden death. N Engl J Med.

[B6] Lauer MS (2001). Exercise electrocardiogram testing and prognosis. Novel markers and predictive instruments. Cardiol Clin.

[B7] Gulati M, Black HR, Shaw LJ, Arnsdorf MF, Merz CN, Lauer MS, Marwick TH, Pandey DK, Wicklund RH, Thisted RA (2005). The prognostic value of a nomogram for exercise capacity in women. N Engl J Med.

[B8] Wei M, Kampert JB, Barlow CE, Nichaman MZ, Gibbons LW, Paffenbarger RSJ, Blair SN (1999). Relationship between low cardiorespiratory fitness and mortality in normal-weight, overweight, and obese men. Jama.

[B9] Ekelund LG, Haskell WL, Johnson JL, Whaley FS, Criqui MH, Sheps DS (1988). Physical fitness as a predictor of cardiovascular mortality in asymptomatic North American men. The Lipid Research Clinics Mortality Follow-up Study. N Engl J Med.

[B10] Cole CR, Blackstone EH, Pashkow FJ, Snader CE, Lauer MS (1999). Heart-rate recovery immediately after exercise as a predictor of mortality. N Engl J Med.

[B11] Anantharam A, Markowitz SM, Abbott GW (2003). Pharmacogenetic considerations in diseases of cardiac ion channels. J Pharmacol Exp Ther.

[B12] Wilde AA, Bezzina CR (2005). Genetics of cardiac arrhythmias. Heart.

[B13] Small KM, McGraw DW, Liggett SB (2003). Pharmacology and physiology of human adrenergic receptor polymorphisms. Annu Rev Pharmacol Toxicol.

[B14] Liggett SB (2003). Polymorphisms of adrenergic receptors: variations on a theme. Assay Drug Dev Technol.

[B15] Imamura A, Okumura K, Matsui H, Mizuno T, Ogawa Y, Imai H, Numaguchi Y, Sakai K, Murohara T (2004). Endothelial nitric oxide synthase and methylenetetrahydrofolate reductase gene polymorphisms are associated with endothelial dysfunction in young, healthy men. Can J Cardiol.

[B16] Puddu P, Cravero E, Puddu GM, Muscari A (2005). Genes and atherosclerosis: at the origin of the predisposition. Int J Clin Pract.

[B17] Tobin MD, Raleigh SM, Newhouse S, Braund P, Bodycote C, Ogleby J, Cross D, Gracey J, Hayes S, Smith T, Ridge C, Caulfield M, Sheehan NA, Munroe PB, Burton PR, Samani NJ (2005). Association of WNK1 Gene Polymorphisms and Haplotypes With Ambulatory Blood Pressure in the General Population. Circulation.

[B18] Bengtsson K, Melander O, Orho-Melander M, Lindblad U, Ranstam J, Rastam L, Groop L (2001). Polymorphism in the beta(1)-adrenergic receptor gene and hypertension. Circulation.

[B19] Poller W, Kuhl U, Tschoepe C, Pauschinger M, Fechner H, Schultheiss HP (2005). Genome-environment interactions in the molecular pathogenesis of dilated cardiomyopathy. J Mol Med.

[B20] Bouchard C, Rankinen T (2001). Individual differences in response to regular physical activity. Med Sci Sports Exerc.

[B21] Bouchard C, Leon AS, Rao DC, Skinner JS, Wilmore JH, Gagnon J (1995). The HERITAGE family study. Aims, design, and measurement protocol. Med Sci Sports Exerc.

[B22] Beckerman J, Wu T, Jones S, Froelicher VF (2005). Exercise test-induced arrhythmias. Prog Cardiovasc Dis.

[B23] Bunch TJ, Chandrasekaran K, Gersh BJ, Hammill SC, Hodge DO, Khan AH, Packer DL, Pellikka PA (2004). The prognostic significance of exercise-induced atrial arrhythmias. J Am Coll Cardiol.

[B24] Evans WE, Relling MV (2004). Moving towards individualized medicine with pharmacogenomics. Nature.

[B25] Kreutz R (2004). Pharmacogenetics of antihypertensive drug response. Curr Hypertens Rep.

[B26] Schwartz GL, Turner ST (2004). Pharmacogenetics of antihypertensive drug responses. Am J Pharmacogenomics.

[B27] Kallio J, Lehtinen R, Viik J, Kööbi T, Niemelä K, Lehtimäki T, Turjanmaa V (2003). Exercise test database analysis software. International Journal of Bioelectromagnetism.

[B28] Mason RE, Likar I (1966). A new system of multiple-lead exercise electrocardiography. Am Heart J.

[B29] Borg GA (1982). Psychophysical bases of perceived exertion. Med Sci Sports Exerc.

[B30] Friedewald WT, Levy RI, Fredrickson DS (1972). Estimation of the concentration of low-density lipoprotein cholesterol in plasma, without use of the preparative ultracentrifuge. Clin Chem.

[B31] Eda S, Kaufmann J, Roos W, Pohl S (1998). Development of a new microparticle-enhanced turbidimetric assay for C-reactive protein with superior features in analytical sensitivity and dynamic range. J Clin Lab Anal.

[B32] Livak KJ (1999). Allelic discrimination using fluorogenic probes and the 5' nuclease assay. Genet Anal.

[B33] Pajunen P, Koukkunen H, Ketonen M, Jerkkola T, Immonen-Räihä P, Kärjä-Koskenkari P, Mähönen M, Niemelä M, Kuulasmaa K, Palomäki P, Mustonen J, Lehtonen A, Arstila M, Vuorenmaa T, Lehto S, Miettinen H, Torppa J, Tuomilehto J, Kesäniemi YA, Pyörälä K, Salomaa V (2005). The validity of the Finnish Hospital Discharge Register and Causes of Death Register data on coronary heart disease. Eur J Cardiovasc Prev Rehabil.

[B34] Lehtinen R, Sievanen H, Viik J, Turjanmaa V, Niemela K, Malmivuo J (1996). Accurate detection of coronary artery disease by integrated analysis of the ST-segment depression/heart rate patterns during the exercise and recovery phases of the exercise electrocardiography test. Am J Cardiol.

[B35] Viik J, Lehtinen R, Malmivuo J (1999). Detection of coronary artery disease using maximum value of ST/HR hysteresis over different number of leads. J Electrocardiol.

[B36] Fletcher GF, Balady G, Froelicher VF, Hartley LH, Haskell WL, Pollock ML (1995). Exercise standards. A statement for healthcare professionals from the American Heart Association. Writing Group. Circulation.

[B37] Gibbons RJ, Balady GJ, Beasley JW, Bricker JT, Duvernoy WF, Froelicher VF, Mark DB, Marwick TH, McCallister BD, Thompson PDJ, Winters WL, Yanowitz FG, Ritchie JL, Cheitlin MD, Eagle KA, Gardner TJ, Garson AJ, Lewis RP, O'Rourke RA, Ryan TJ (1997). ACC/AHA Guidelines for Exercise Testing. A report of the American College of Cardiology/American Heart Association Task Force on Practice Guidelines (Committee on Exercise Testing). J Am Coll Cardiol.

[B38] Chen G, Redberg RF (2000). Noninvasive diagnostic testing of coronary artery disease in women. Cardiol Rev.

[B39] Mora S, Redberg RF, Cui Y, Whiteman MK, Flaws JA, Sharrett AR, Blumenthal RS (2003). Ability of exercise testing to predict cardiovascular and all-cause death in asymptomatic women: a 20-year follow-up of the lipid research clinics prevalence study. Jama.

[B40] Kurl S, Laukkanen JA, Rauramaa R, Lakka TA, Sivenius J, Salonen JT (2001). Systolic blood pressure response to exercise stress test and risk of stroke. Stroke.

[B41] Viik J, Lehtinen R, Turjanmaa V, Niemela K, Malmivuo J (1997). The effect of lead selection on traditional and heart rate-adjusted ST segment analysis in the detection of coronary artery disease during exercise testing. Am Heart J.

[B42] Lehtinen R, Sievanen H, Viik J, Vuori I, Malmivuo J (1997). Reproducibility of the ST-segment depression/heart rate analysis of the exercise electrocardiographic test in asymptomatic middle-aged population. Am J Cardiol.

[B43] Viik J, Lehtinen R, Turjanmaa V, Niemela K, Malmivuo J (1998). Correct utilization of exercise electrocardiographic leads in differentiation of men with coronary artery disease from patients with a low likelihood of coronary artery disease using peak exercise ST-segment depression. Am J Cardiol.

[B44] Nikus KC, Eskola MJ, Niemela KO, Sclarovsky S (2005). Modern morphologic electrocardiographic interpretation--a valuable tool for rapid clinical decision making in acute ischemic coronary syndromes. J Electrocardiol.

[B45] Nordlie MA, Wold LE, Kloner RA (2005). Genetic contributors toward increased risk for ischemic heart disease. J Mol Cell Cardiol.

[B46] Winkelmann BR, Hager J (2000). Genetic variation in coronary heart disease and myocardial infarction: methodological overview and clinical evidence. Pharmacogenomics.

